# Medical Items

**Published:** 1895-07

**Authors:** 


### *MEDICAL ITEMS.*

WE learn that one of the firm of the Amick Cure concern has lately died of consumption.

THE Cincinnati College of Medicine and Surgery has become the Medical Department of the University of Cincinnati.

NOTICE.—Our offer of a thermometer to each cash-in-advance subscriber is withdrawn. *New* subscribers, paying full year in advance, may still get the thermometer. Book offer still open to all.

DR. W. A. CROW, of Atlanta, sailed for Europe on the 12th ult. He will spend several months in Berlin, Munich, Vienna, Paris, London, and Birmingham, in hospitals of obstetrics and gynecology.

THE death-rate of the world's population is said to be 68 per minute, and the birth-rate 70 per minute. Thus the birth-rate seems to have it by about 3,000 per day, or 1,095,000 per year. Evidently there is life in the old world yet.

THE Association of American Medical Editors elected officers for the ensuing year as follows: Dr. George M. Gould, of Philadelphia, President; Dr. Ohmann-Dumesnil, St. Louis, Treasurer, and Dr. H. B. Ellis, of Los Angeles, Cal., Secretary.

THE American Medical Publishers' Association elected the following officers: President, Dr. Landon B. Edwards, of Richmond; Vice-President, Dr. J. C. Culbertson, of Cincinnati, Ohio; Treasurer, J. MacDonald, Jr., of New York City; Secretary, Charles W. Fassett, of St. Joseph, Mo.

A KANSAS court says that Keeley must show up. A gentleman sues Keeley for $100,000 damages because, he alleges, the "gold cure" has made him a physical wreck. The court rules that as Keeley's method is not protected by patent he must tell the nature and ingredients of his so-called bichloride-of-gold compound.

---

THE American Medical College Association have decided that the four-years' course shall go into effect with the students entering in 1895. The following officers were elected: President, Dr. William Osler of Baltimore; Vice-Presidents, Drs. J. M. Bodine of Louisville, and D. W. Graham of Chicago; Secretary and Treasurer, Dr. Bayard Holmes of Chicago; and Judicial Council, Drs. D. S. Reynolds of Louisville, W. H. Pancoast of Philadelphia, and Victor Vaughan of Ann Arbor, Mich.

---

WELL, this is a joke! Here is our lovely contemporary, the Philadelphia *Times and Register*, prating about the bad habit of some journals abstracting and purloining articles from others without the credit of a reference (issue June 1st, page 448). To which we heartily subscribe. However, if the editor will look at the last item on page 456, same issue, he will find some matter which was lifted bodily from the May number of this JOURNAL without the slightest "credit of a reference." The *Times and Register* should sweep before its own door.

---

NOT A UNIQUE CASE.—The curtain has risen on the third act, and the momentary hush that preceded the resumption of the performance on the stage was broken by a stentorian voice from the rear of the auditorium: "Is Dr. Higginspiker in the house?" A tall, heavily-whiskered man occupying a front seat rose up. "If Dr. Higginspiker is in the house," resumed the stentorian voice, "he told me I was to come here and call him out at 10 o'clock!"

Whereupon Dr. Higginspiker, looking very red, picked up his hat and cane and walked down the aisle amid much loud and enthusiastic applause.—*Chicago Tribune.*

---

SOME discussion has arisen over the effort of one of the leading "old line" insurance companies to reduce the examiner's fee from five to three dollars. Examiners certainly have the right to object, considering the work done and the value of the service to the company. The *Atlantic Medical Weekly* wisely remarks: "The examiners' blanks have increased in size and number of questions, until what was fifteen years ago the task of a few moments is now one of an hour, to say nothing of the time required for urinary analysis. If the company wishes industrial insurance, it can probably get examiners for fifty cents, and thus be able to add a little more to the meager salary of the president."

---

THE unrestricted traffic in patent medicines is a disgrace to our civilization. Communities and States are up in arms against alcohol as a beverage in all its forms except the one most detestable and villainous. Introduce some vile ingredient into the alcohol, christen and label it as a patent medicine, and lo! it is robed in innocence and welcomed to the homes of the righteous. A poison can be legally sold as such in most States only with a flaring label of warning, and after double and triple forms of registration by a competent pharmacist. Put it up as a patent medicine, decorate it with a seductive title and a rainbow wrapper, and at once it is beyond the restraints of regulation, and may be sold by street-hawkers, slaughter shops, anybody and everybody, without let or hindrance. Where is the consistency, the intelligence, the justice in this strange partiality for the nostrum curse?—*Western Druggist.*

